# Vasopressin Receptor Antagonists in Hyponatremia: Uses and Misuses

**DOI:** 10.3389/fmed.2017.00141

**Published:** 2017-08-21

**Authors:** Helbert Rondon-Berrios, Tomas Berl

**Affiliations:** ^1^Department of Medicine, University of Pittsburgh, Pittsburgh, PA, United States; ^2^Department of Medicine, University of Colorado, Aurora, CO, United States

**Keywords:** vasopressin, hyponatremia, vasopressin antagonists, vaptans, osmotic demyelination syndrome

## Abstract

Decreases in the concentration of sodium in plasma constitute hyponatremia, the commonest electrolyte disorder in clinical medicine. It is now well established that its presence conveys an increased mortality risk even when the decrement is mild. In addition, recent evidence suggests that chronic and apparently asymptomatic hyponatremia is associated with increased morbidity including neurocognitive deficits and bone fractures. Furthermore, hyponatremia is associated with higher health care-related expenses. Consequently, exploring new therapeutic strategies that increase plasma sodium in a safe and effective manner is of paramount importance. In this regard, there are scant data to support the use of traditional management strategies for hyponatremia (fluid restriction, salt tablets, loop diuretics, and normal saline). Furthermore, data from a large hyponatremia registry reveal the limited efficacy of these therapies. More recently vasopressin receptor antagonists provide a promising treatment for hyponatremia by targeting its most common mechanism, namely, increased vasopressin activity. However, uncertainty still lingers as to the optimal indications for the use of vasopressin receptor antagonists in hyponatremia and a few reports have described complications resulting from their misuse. This review summarizes the appropriate and inappropriate uses of vasopressin receptor antagonists in the treatment of hyponatremia.

## Introduction

The posterior pituitary hormone vasopressin has a primordial role in the pathogenesis of hyponatremia, the commonest electrolyte disorder in inpatients (1–3). Furthermore, studies consistently find an excess morbidity and mortality in patients with hyponatremia compared to patients with normal plasma sodium concentration (PNa). The association has been reported in hospitalized patients (3, 4) in intensive care units (5, 6), and in apparently asymptomatic outpatients with much less severe forms of hyponatremia (7). The association of hyponatremia with increased mortality holds true in numerous clinical settings including heart failure (8), cirrhosis (9), cancer (10), orthopedic surgery (11), chronic kidney disease (12), and even end-stage renal disease (13, 14). The conflicting guidelines issued by two expert panels (15, 16), brings to light uncertainties that remain as to the optimal therapeutic approach to this clinical entity. This confusion is a consequence of the dearth of randomized controlled trials (RCTs), and the lack of data supporting the safety and reliability of one approach over the others. Furthermore, data from the largest hyponatremia registry reveal the limited efficacy of traditional therapeutic approaches (fluid restriction, salt tablets, loop diuretics, and normal saline) (17, 18). In this setting, the development and now availability of drugs that antagonize the action of vasopressin, and by that directly addressing the most common pathogenesis of this disorder, is a most welcome addition to the therapeutic arsenal of hyponatremia. This review summarizes the appropriate and inappropriate uses of these antagonists on the background of the larger studies and meta-analyses that have analyzed their efficacy and potential side effects.

## Appropriate Uses of VRAs

### Clinical Vignette #1

A 67-year-old man presents to the emergency department complaining of severe low back pain for 2 days. He has a past medical history of tobacco use for 40 years and recent diagnosis of extended-stage small cell lung cancer (SCLC) with spinal metastases currently undergoing first cycle of chemotherapy with etoposide and cisplatin. On exam, he appears in mild distress from pain. Vital signs reveal a blood pressure of 150/70 mmHg and heart rate of 102 bpm. His weight is 65 kg. His urine output is 25 mL/h. No jugular venous distention. Cardiopulmonary and abdominal examinations are unremarkable. Patient has tenderness to palpation over the spinous processes of L3 and L4 vertebrae. Neurological exam shows an alert and oriented patient without any focal neurological deficits. Laboratory examination reveals the following blood chemistries: PNa = 112 mmol/L, K = 3.8 mmol/L, Cl = 78 mmol/L, TCO_2_ = 26 mmol/L, BUN = 2.86 mmol/L (8 mg/dL), creatinine = 79.56 μmol/L (0.9 mg/dL), glucose = 5.66 mmol/L (102 mg/dL), plasma osmolality = 235 mmol/kg (235 mOsm/kg), cortisol = 800.05 nmol/L (29 µg/dL), TSH = 3.8 mIU/L, and uric acid = 190.35 μmol/L (3.2 mg/dL). Urine tests reveal the following: urine osmolality = 645 mmol/kg (645 mOsm/kg), UNa = 170 mmol/L, and UK = 35 mmol/L. MRI of the lumbar spine demonstrates the presence of new vertebral metastases over L3, L4, and L5 without evidence of spinal cord compression.

After excluding other causes, patient’s hyponatremia is attributed to the syndrome of inappropriate antidiuretic hormone secretion (SIADH) and he is treated with the combination of fluid restriction of less than 1,500 mL/day and urea 30 g by mouth twice a day, which results in an increase of PNa to 116 mmol/L at day 2 of hospitalization. Unfortunately patient is unable to tolerate oral urea due to poor palatability. A decision is then made to switch to tolvaptan 15 mg by mouth once a day. After 4 h of initial tolvaptan administration, urine output increases to 160 mL/h and urine osmolality decreases to 150 mmol/kg (150 mOsm/kg). PNa then increases to 123 mmol/L. In view of the fact that PNa goal of correction is achieved and aware of the risk of overcorrection in the face of large water diuresis then an infusion of D5W is initiated at a rate to match urine output and avoid further correction until next day. The next day, D5W is discontinued and PNa is allowed to increase further to 130 mmol/L (130 mEq/L). Along with these measures, an optimal pain regimen is instituted. Patient is finally discharged home at day 4 of hospitalization on tolvaptan with monthly evaluation of liver function tests.

VRAs can be appropriately used in use in the treatment of non-severe hyponatremia such as patients without severe symptoms of hyponatremia or PNa ≥ 120 mmol/L (120 mEq/L) caused by SIADH or heart failure especially if fluid restriction and other therapies have failed (see Table 1).

**Table 1:** Appropriate uses of VRAs.

Hyponatremia from syndrome of inappropriate antidiuretic hormone secretion Malignancy, especially small cell lung cancer^a^ Intracranial disorders Pulmonary disorders Medications, when chronic use is required Nausea or pain, when chronic and intractable Idiopathic Hyponatremia from heart failure Non-severe hyponatremia Hyponatremia that is not amenable to correction with fluid restriction or other therapies

*^a^Strong supporting data*.

#### Use of VRAs in Hyponatremia due to SIADH or Heart Failure

Vasopressin-mediated decreased renal free water excretion constitutes the most common mechanism of hyponatremia in clinical medicine. In this context, vasopressin could be released under conditions associated with decreased effective arterial blood volume from hypovolemia, heart failure, or cirrhosis; SIADH, or cortisol deficiency. SIADH is a form of euvolemic hypotonic hyponatremia that results from inappropriate and continuous secretion of vasopressin despite normal plasma volume and is considered the most common cause of hyponatremia (19). SIADH has multiple etiologies including intracranial disorders, pulmonary disorders, medications, nausea, and pain. Malignancy is the commonest causes of SIADH. VRAs are indicated in the treatment of hyponatremia of SIADH and heart failure (see Table 1). VRAs are indicated in SIADH when fluid restriction is likely to fail, is not tolerated, or fails to increase PNa. The efficacy of VRAs in the hyponatremia of heart failure and SIADH has been well established in the literature. To this end, landmark trials, meta-analyses, and the observations of a large hyponatremia registry appraising the efficacy and safety of oral tolvaptan or intravenous conivaptan are herein briefly described.

The SALT-1 and SALT-2 were two identical RCTs; one in Europe and one in the Americas, which enrolled 448 patients with euvolemic and hypervolemic hyponatremia PNa < 135 mmol/L to tolvaptan or placebo and followed them for 30 days (20). SIADH, heart failure, and cirrhosis were the most common etiologies. These trials excluded patients with severe hyponatremia (presence of severe symptoms or PNa < 120 mmol/L). PNa at days 4 and 30 was significantly higher in the tolvaptan arm. Patients in the tolvaptan group discontinued the drug at the end of the trials and hyponatremia recurred in most of them. Adverse events occurred at similar rates in the placebo and the experimental groups in the SALT trials. The majority of adverse events were related to the aquaretic properties of tolvaptan including xerostomia, and increased thirst and urination. Overcorrection of hyponatremia occurred only in 1.8% of patients in the SALT trials.

The SALTWATER study (21) constituted the follow up of the SALT trials enrolling 111 patients and followed them for a median of 1.9 years. All patients received tolvaptan that was titrated up to reach a normal PNa. Fifty-seven percent of patients achieved normal PNa, which was maintained during the study. Tolvaptan has been shown to have an overall acceptable safety profile in these two studies. Only six patients in the SALTWATER study discontinued the study drug due to the aquaretic adverse events of tolvaptan described above.

Since SIADH is one of the main indications for VRA use, and malignancy is the commonest cause of SIADH (22), it is fitting to mention some details about the use of VRAs in cancer-associated SIADH. At least 70% of cases of cancer-associated SIADH are caused by SCLC (23). On the other side, up to 15% of patients with SCLC develop SIADH (24). There is solid evidence supporting the efficacy of VRAs in cancer-associated SIADH. Salahudeen et al. (25) conducted an RCT in which the investigators randomized patients with cancer with non-hypovolemic hyponatremia to either placebo or tolvaptan. Patients in both groups were allowed to “drink-to-thirst.” The primary outcome was PNa correction by day 14. Secondary outcomes included length of hospital stay and change in Mini-Mental State Examination scores. The data safety monitoring board stopped the study after randomizing 30 patients when the primary endpoint was reached (94% in tolvaptan group vs. 8% in placebo group), which met study stopping rule for superiority. The secondary endpoints were statistically non-significant between the two groups. Gralla et al. (26) performed a *post hoc* analysis of the SALT trials that analyzed 28 hyponatremic patients with SIADH and cancer. The most common causes of cancer were lung (29%), head and neck (25%), breast (11%), and renal (11%). Patients in the tolvaptan group compared to placebo showed a highly significant improvement in PNa by day 4 (5 vs. −0.3 mmol/L) and by day 30 (6.9 vs. 1 mmol/L). Some case reports and case series describe peculiarities in the use of VRAs in cancer-associated SIADH. Kenz et al. (27) reported a case series of 13 patients with paraneoplastic SIADH in 7 patients with SCLC and 6 patients with other malignancies. An initial single dose of 15 mg of tolvaptan overcorrected hyponatremia in two patients prompting the clinicians to reduce the dose to 7.5 mg every other day in the rest of the patients, which corrected and maintained a normal PNa in the majority. This observation was interpreted as reflecting a higher sensitivity to tolvaptan in patients with paraneoplastic SIADH. This observation should be confirmed by further studies. Correction and stabilization of PNa are usually required for initiation of chemotherapy in patients with cancer. Petereit et al. (28) reported the results of a prospective cases series of 10 patients with advanced stage SCLC with hyponatremia due to SIADH. Employing an algorithmic approach, patients were treated with tolvaptan 15 mg daily leading to optimal correction of hyponatremia with a median duration of treatment of 4 days, which led to an improvement in performance status ensuring prompt initiation of chemotherapy in all patients. Cell lines of SCLC have been shown to produce vasopressin *in vitro*, and this constitutes one of the main mechanisms by which SCLC causes SIADH (i.e., ectopic production) (29). Saintigny et al. (30) reported a case of a patient with SCLC who developed SIADH associated with massive release of vasopressin from tumor cells, as demonstrated by the presence of very high levels of vasopressin immediately after each of the first two cycles of chemotherapy, resulting in chemotherapy-induced tumor lysis syndrome. This case report might suggest a potential role for prophylactic use of VRAs before initiation of chemotherapy in selected patients with SCLC.

Zeltser et al. (31) randomized 84 patients with euvolemic or hypervolemic hyponatremia with PNa ranging from 115 to 130 mmol/L to two different doses of intravenous conivaptan (40 and 80 mg) or placebo for 4 days. SIADH and heart failure were the commonest causes of hyponatremia in this population. Conivaptan, regardless of the dose, was associated with a significant increase in PNa. No difference in PNa was found between conivaptan doses. The rate of adverse events in both groups was similar. Nevertheless, conivaptan was associated with a higher rate incidence of phlebitis and other infusion-site reactions especially when the 80 mg dose was used. Other adverse events attributed to conivaptan were hypotension and renal dysfunction. None of these led to discontinuation of the drug. Overcorrection of hyponatremia occurred in 7% of patients on conivaptan in this study.

Five meta-analyses have also been published up to this date establishing the efficacy of VRAs in the treatment of hyponatremia.

Rozen-Zvi et al. (32) evaluated 15 studies produced from 1999 to 2009 assessing the short-term effects of all VRAs combined in the treatment of euvolemic or hypervolemic hyponatremia involving 1,125 patients. VRAs were associated with a greater early response rate of PNa than controls (RR = 3.15, 95% CI: 2.27–4.37) but with significant heterogeneity (*I*^2^ = 55%). A risk of rapid PNa correction with an RR of 2.52 (95% CI: 2.56–4.41) was found in eight trials.

Jaber et al. (33) analyzed 11 RCTs published between 2003 and 2009 examining the success of all VRAs combined in the treatment of euvolemic and hypervolemic hyponatremia involving 1,094 patients. A Jadad score of 3–4 indicated good quality of the trials in the meta-analysis. VRAs were associated with a significant rise in PNa of 3.3 mmol/L at day 1 (95% CI: 2.7–3.8, *P* < 0.001) without significant heterogeneity (*I*^2^ = 0%, *P* = 0.51). This meta-analysis also found an increase of rapid PNa correction with an OR of 3.0 (*P* < 0.001) in nine trials.

Zhang et al. (34) conducted a meta-analysis of 18 RCTs published between 2003 and 2014 that examined the efficacy of all VRAs in the treatment of euvolemic and hypervolemic hyponatremia appraising 1,806 patients. The quality of the studies was good as indicated by a Jadad score of 2–5. Use of VRAs resulted in a significant net increase in PNa of 4.89 mmol/L (95% CI: 4.35–5.43) in the random effects model and 4.70 mmol/L (95% CI: 4.45–4.95) in the fixed effects model. The heterogeneity was significant (*I*^2^ = 67.2%). No publication bias was found in the assessment (*P*_Eager_ = 0.45). An increased risk of rapid PNa correction was also found in 12 studies of this meta-analysis with an RR = 2.56 (95% CI: 1.45–4.53).

Li et al. (35) carried out the analysis of 11 studies published between 2008 and 2016 that examined the efficacy of tolvaptan in the treatment of hyponatremia covering 5,209 patients. Nine studies reported the changes in PNa after tolvaptan therapy, and significant heterogeneity was observed across these studies (*I*^2^ = 76%, *P* < 0.001). Therefore, a random effect model was used. The use of tolvaptan was found to be associated with an increase in PNa of 3.99 mmol/L (95% CI: 2.80–5.19, *Z* = 6.56, *P* < 0.001). Publication bias was not found. The use of tolvaptan was also found to be associated with a rapid PNa correction (RR = 8.43, 95% CI: 1.06–66.96, *Z* = 2.02, *P* = 0.04) in two trials.

Bhandari et al. (36) performed a meta-analysis of 18 trials published between 2003 and 2014 that examined the effectiveness of VRAs in hyponatremia and included a total of 3,408 patients in their analysis. The analysis suggests that patients randomized to a VRA (except conivaptan) are significantly more likely to normalize PNa and/or increase it by more than 5 mmol/L with tolvaptan having the largest effect (RR = 3.3, 95% CI: 1.97–5.54). However, pooled analysis of tolvaptan trials showed a statistically significant risk of rapid PNa correction (RR = 9.85, 95% CI: 1.27–76.35, *P* = 0.03) with uncertain treatment effect size given low number of events in each individual trial.

The efficacy of tolvaptan is also supported by the analysis of the largest hyponatremia registry to this date with over 3,000 patients describing the therapeutic practice patterns regarding hyponatremia in the United States and Europe (17, 18). In this registry, the rise in PNa (interquartile range) during first 24 h was greater with tolvaptan compared to other traditional therapies. Similar results were observed in the analysis of a registry of cancer patients with euvolemic hyponatremia (37).

In summary, several trials and meta-analyses support the efficacy and safety of oral tolvaptan and intravenous conivaptan in the treatment of hyponatremia. The results of these trials show a great uniformity with remarkably similar changes in PNa. Although the SALT trials included patients with cirrhosis, recent data cautions about their use (see Inappropriate Uses of VRAs). All five meta-analyses of VRAs also reaffirm their efficacy with similar changes in PNa although significant heterogeneity was found in the majority of them. Furthermore, all meta-analyses showed a risk of rapid PNa correction with VRA use, and rarely cases of osmotic demyelination syndrome (ODS) have been reported in association with these drugs (38). Therefore, preventive measures need to be in place (i.e., D5W infusion to match urine output once goal of PNa correction is achieved) to avoid overcorrection of hyponatremia. Long-term use of VRAs remains necessary in certain patients (e.g., SIADH from malignancy) as hyponatremia will recur upon discontinuation of the drug as observed in the SALT trials. In these cases, administration of standard doses of tolvaptan (15–60 mg) and periodic liver function test monitoring are strongly recommended (39).

## Inappropriate Uses of VRAs

### Clinical Vignette #2

A 71-year-old woman with a past medical history of systemic hypertension and osteoarthritis is brought to the emergency department after a mechanical fall. Her family reports that her blood pressure had been difficult to control and hydrochlorothiazide 25 mg daily was added about 1 week ago. On exam, she appears confused and mildly agitated. Vital signs in supine position reveal a blood pressure of 140/75 mmHg and heart rate of 102 bpm. Vital signs in upright position demonstrate a blood pressure of 90/56 mmHg and heart rate of 120 bpm. Her weight is 45 kg. Jugular veins are flat. Cardiopulmonary and abdominal examinations are unremarkable. There is tenderness to palpation over the left hip. Neurological exam shows a confused and disoriented frail elderly woman without any focal neurological deficits. Laboratory examination reveals the following blood chemistries: Na = 110 mmol/L, K = 3.4 mmol/L, Cl = 78 mmol/L, TCO_2_ = 26 mmol/L, BUN = 12.14 mmol/L (34 mg/dL), Cr = 61.88 µmol/L (0.7 mg/dL), glucose = 5.49 mmol/L (99 mg/dL), and plasma osmolality = 239 mmol/kg (239 mOsm/kg). Urine tests reveal the following: urine osmolality = 345 mmol/kg (345 mOsm/kg), UNa = 21 mmol/L, and UK = 35 mmol/L. Pelvic X-ray shows a minimally displaced left inferior pubic ramus fracture.

She is initially treated with hypertonic saline (NaCl 3%) slow infusion for her symptomatic hyponatremia. However, her PNa fails to rise, and she is initiated on tolvaptan 15 mg oral daily. On the subsequent day, her PNa increases to 139 mmol/L. Her overall condition improves, and she is discharged to a rehabilitation facility after 2 days.

She presents again to the emergency room a week later with aphasia and quadriparesis. Head CT is negative for acute intracranial pathology but a brain MRI reveals high-signal intensity on T2-weighted images in the mid pons suggestive of central pontine myelinolysis.

The use of VRAs is considered inappropriate in a number of settings (see Table 2).

**Table 2:** Inappropriate uses of VRAs.

Contraindicated Hypovolemia ▪ Gastrointestinal losses: vomiting, diarrhea, and bleeding ▪ Skin losses: burns and excessive sweating ▪ Third spacing: acute pancreatitis and small bowel obstruction ▪ Renal losses: diuretic use, aldosterone deficiency, cerebral salt wasting syndrome, and salt wasting nephropathies Severe hyponatremia (presence of severe symptoms or PNa < 120 mmol/L) History of hypersensitivity reaction to vasopressin receptor antagonists Anuria Impaired thirst mechanism Concomitant use of drugs that increase plasma levels of vasopressin receptor antagonists (i.e., CYP3A4 inhibitors)
Strongly advise against its use Liver disease^a^ Concomitant use of drugs that increase plasma sodium concentration (e.g., hypertonic saline)
Ineffective (low ADH states) Primary polydipsia Low solute intake GFR < 10 mL/min Nephrogenic syndrome of inappropriate antidiuresis (NSIAD)
Unnecessary (transient high ADH states) Cortisol deficiency Thyroid hormone deficiency Drug-induced syndrome of inappropriate antidiuretic hormone secretion

*^a^A short-course of VRAs are used sometimes in cirrhotic patients with significant hyponatremia awaiting liver transplantation in an attempt to expedite surgery*.

#### Hypovolemic Hyponatremia

VRAs are contraindicated in hyponatremia due to hypovolemia as its use may exacerbate hypotension (16). Hypovolemia can be caused by gastrointestinal losses such as vomiting, diarrhea, and bleeding; skin losses such as excessive sweating and burns; third spacing such as small bowel obstruction and acute pancreatitis; and renal losses such as use of diuretics, salt wasting nephropathy, aldosterone deficiency, and cerebral salt wasting syndrome (CSWS). CSWS deserves a special mention as this disorder shares many clinical features of SIADH (40). Hyponatremia in CSWS appears in the context of an intracranial pathology, typically subarachnoid hemorrhage, where either an impaired sympathetic input or an unidentified brain natriuretic peptide associated with the intracranial disorder causes renal sodium wasting. As in SIADH, patients with CSWS have also a hypotonic hyponatremia associated with high urine osmolality and high urine sodium, but they are actually hypovolemic from sodium wasting. This could be manifested as frank hypotension or tachycardia, or more subtly, as hemoconcentration, hyperalbuminemia, or an elevated BUN/creatinine ratio (40). VRAs are not indicated in this disorder. Volume expansion should be the primary goal in this setting. Nevertheless, some experts have questioned the existence of CSWS arguing not only that there is no gold standard to clinically assess volume status (the main differentiating key clinical feature between SIADH and CSW) but also, that given the risk for cerebral edema, all patients with hyponatremia with intracranial pathology should be treated with hypertonic saline independent of the cause (41). Furthermore, CSWS seems to be a rare entity as most cases of hyponatremia in the context of subarachnoid hemorrhage are either due to SIADH or cortisol deficiency (42, 43).

#### Severe Hyponatremia

Trials on VRAs excluded patients with severe symptoms of hyponatremia where urgent treatment is indicated since VRA onset of action is delayed for 2–4 h. Patients with PNa < 120 mEq/L were also excluded from these trials.

#### Concomitant Use of Certain Drugs

VRAs are metabolized *via* CYP3A4 cytochrome therefore VRAs can interact with its inhibitors (e.g., clarithromycin, fluconazole, and diltiazem). Dose adjustment is a requirement when these drugs are used in combination. Conivaptan is considered a strong inhibitor of CYP3A4, and for this reason, its use has been limited to 4 days of intravenous administration (44).

#### Liver Disease

Concerns for liver damage associated with VRAs appeared in the TEMPO 3:4 trial (45) studying the efficacy and safety of tolvaptan to slow down the progression of autosomal dominant polycystic kidney disease (ADPKD). No elevation of liver function tests was observed in the SALT and SALTWATER trials. It is important to point out that the tolvaptan doses used in the TEMPO 3:4 trial were much higher than the doses commonly used to treat hyponatremia. A *post hoc* analysis study (46) demonstrated that these events are rare. Nevertheless, the FDA issued a drug safety communication (47) restricting the use of tolvaptan to 30 days and avoiding its use in patients with underlying liver disease. A recent press release from Otsuka, the manufacturer of tolvaptan, announced the results of a phase 3 trial of patients with ADPKD using high doses of tolvaptan (45–120 mg/day) (48) and indicated that tolvaptan resulted in more patients than placebo with increased transaminases, but none of these patients exhibited total bilirubin greater than two times the upper limit of normal and therefore did not meet Hi’s criteria for drug-induced liver injury.

Being a dual V1a/V2 receptor antagonist, conivaptan is also contraindicated in cirrhosis as blockage of V1a receptor effects can trigger splanchnic vasodilation and subsequent hypotension, hepatorenal syndrome, or variceal bleeding (49).

A special situation that deserves mention here is the use of VRAs in cirrhotic patients who are awaiting liver transplantation. These patients are at increased risk of ODS after transplantation due to rapid increase in PNa in the immediate postoperative period (50, 51). This heightened risk of complications constitutes a real concern for many transplant surgeons who might delay a life-saving surgery until PNa is corrected to an acceptable level. Therefore, some have advocated the short-term use of VRAs in an attempt to expedite liver transplant surgery (52). However, there are no data or consensus in this regard and other groups have discouraged their use (53).

#### Vasopressin-Independent Hyponatremia

VRAs are not indicated, nor would they be expected to be effective in the treatment of hyponatremia that is not mediated by vasopressin such as primary polydipsia and low solute intake (39). VRAs are also ineffective in patients with the nephrogenic syndrome of inappropriate antidiuresis (NSIAD), a gain-of-function mutation of the V2 receptor causing and characterized by low vasopressin levels. Urea, which enhances water excretion by a different mechanism, however (54), has been used successfully in this entity (55).

#### Hyponatremia due to Low GFR

VRAs have a very limited efficacy in patients with hyponatremia from low GFR due to poor fluid delivery to the distal diluting nephron segments.

#### Hyponatremia due to Transient High Vasopressin States

VRAs should not be used in transient high vasopressin states such as cortisol deficiency or drug-induced SIADH. Steroid replacement or discontinuation of the offending agent in these settings is more appropriate therapeutic options.

## Remaining Uncertainties about the Use of VRAs in Hyponatremia

Despite their efficacy and relative safety, some uncertainties remain with the use of VRAs in hyponatremia. A central issue underlying the uncertainty is the absence of cost-effectiveness data. Justifying the high cost of these drugs is challenging given the absence of positive outcome data (56) particularly because benefits on clinically meaningful outcomes or on health-related expenses are lacking. Up to this date, no studies have equivocally shown a decrement in morbidity and mortality as a result of the use of VRAs (39). However, there are potentially encouraging signals in the literature that we will briefly describe.

### Effects of VRAs on Morbidity

Recent evidence suggests that mild or asymptomatic chronic hyponatremia is associated with increased risk of morbidity outcomes such as neurocognitive deficits and gait abnormalities (7). In this regard, the mental component of the SF-12 survey of general health was significantly improved in the combined analysis of both SALT trials and in the SALT-1 trial but not in the SALT-2 trial (20).

The INSIGHT study (57) was a multicenter randomized double-blinded pilot study aimed to investigate the effects of tolvaptan on hyponatremia morbidity outcomes in 57 adult patients age 50 and older with chronic and apparently asymptomatic hyponatremia PNa 120–135 mmol/L. Patients were randomized to uptitrating doses of tolvaptan or placebo for 3 weeks. The primary endpoint was change in neurocognitive composite scores of speed domains (reaction time, psychomotor speed and processing speed). Mean baseline scores were found to be more than 1 SD lower compared to age-matched normal controls. Use of tolvaptan was associated with a statistically significant increase in PNa (from 129 to 136 mEq/L) compared to placebo (from 130 to 132 mEq/L) (*P* < 0.001). There was no difference in the primary outcome. However, tolvaptan use was associated with a statistically significant improvement of score in the psychomotor speed domain (treatment effect, 0.27; 95% CI: 0.04–0.51; *P* = 0.03).

### Effects of VRAs on Mortality

The ACTIV in CHF trial (58) randomized 319 hospitalized patients with heart failure ejection fraction of less than 40% with refractory congestive symptomatology to tolvaptan or placebo. Despite a significant weight loss associated with tolvaptan, rates of worsening heart failure were not different than placebo. *Post hoc* analysis of this trial (59) showed decreased mortality at 60 days associated with improvement of PNa with tolvaptan use. Subsequently, the EVEREST outcome trial (60) randomized 4,133 hospitalized patients with heart failure to tolvaptan or placebo. Primary outcomes were all-cause mortality and a combined endpoint of cardiovascular mortality or subsequent hospitalization for worsening heart failure. Only 11.4% of patients had hyponatremia at baseline in this trial. There were no significant differences in the primary outcomes between groups. A subgroup analysis of this trial showed that tolvaptan was associated with a significant reduction in cardiovascular morbidity and mortality after discharge (61).

### Effects of VRAs on Length of Hospital Stay and Other Health Care-Related Expenses

Dasta et al. performed a study to evaluate the potential cost savings associated with tolvaptan usage in SIADH based on the SALT trials. Use of tolvaptan was associated with a reduced hospital stay and an estimated mean hospital cost reduction of $694 per admission in the United States (62).

Cyr et al. conducted a secondary analysis of the SALT trials and showed that hyponatremic patients who received tolvaptan had a hospital stay that was 1.72 days shorter compared to placebo but this not reached statistical significance (63).

Lee et al. (64) constructed a decision-analytic model using the Korean National Health Insurance database to measure the financial impact of tolvaptan compared with placebo in patients with euvolemic and hypervolemic hyponatremia during a 1-month treatment period. The analysis revealed that tolvaptan was more efficacious with less associated costs in patients with marked hyponatremia.

Jamookeeah et al. (65) performed a cost-utility analysis using a discrete event simulation to model the progression of patients with hyponatremia due to SIADH who have failed fluid restriction (or were not suitable to fluid restriction) and were therefore treated with tolvaptan or no active treatment through inpatient admissions over a 30-day period in Sweden. Tolvaptan use was associated with reduced costs and increased quality-adjusted life years compared with no active treatment strategy.

### Guideline Recommendations Regarding Use of VRAs in Hyponatremia

American and European panels have released opposing guidelines regarding the use of VRAs in hyponatremia. The American expert panel (16) recommended the use of VRAs in non-severe hyponatremia in the setting of SIADH, cirrhosis, and heart failure when fluid restriction is unsuccessful. The European panel (15) does not support the use of VRAs in SIADH and discourages their use in heart failure and cirrhosis citing lack of hard outcome benefits as well as liver injury concerns and meta-analysis signals for PNa overcorrection.

## Conclusion

More than a decade after vasopressin receptor antagonists were approved for clinical use in the management of euvolemic and hypervolemic hyponatremia their use in the treatment of this most common electrolyte disorder remains limited and tentative. This is despite an absence of serious toxicity and despite their highly predictable efficacy as they target the most common underlying mechanism of hyponatremia more directly than any of the existing alternative treatments. This reluctance is most likely related to concerns of overcorrection causing ODS and to the high cost of the drug while data on its impact on solid end points such as cardiovascular events, hospitalizations, and death are lacking. ODS has been reported only very rarely and when VRAs are inappropriately used such as the concomitant administration of hypertonic saline as illustrated in the presented case. Concomitantly data should be collected and studies undertaken to ascertain whether the correction of hyponatremia abated the increased risk for mortality that characterizes this electrolyte disturbance.

## Author Contributions

HR-B has written the sections of appropriate and inappropriate uses of vasopressin antagonists. TB has written the introduction and abstracts. Both HR-B and TB contributed equally to developing the tables and conclusions.

## Conflict of Interest Statement

The authors declare that the research was conducted in the absence of any commercial or financial relationships that could be construed as a potential conflict of interest. The reviewer TC and handling Editor declared their shared affiliation.
